# Quantitative prediction of postpartum hemorrhage in cesarean section on machine learning

**DOI:** 10.1186/s12911-024-02571-7

**Published:** 2024-06-13

**Authors:** Meng Wang, Gao Yi, Yunjia Zhang, Mei Li, Jin Zhang

**Affiliations:** 1 School of Information Engineering, China University of Geosciences, Beijing, 100083 China; 2 Tangshan Polytechnic College, Tangshan, 063299 China; 3 Shijiazhuang Obstetrics and Gynecology Hospital, Shijiazhuang, 050000 China

**Keywords:** Postpartum hemorrhage, Regression, Machine learning, Cesarean section, Random forest, Permutation importance, Partial dependence plot

## Abstract

**Background:**

Cesarean section-induced postpartum hemorrhage (PPH) potentially causes anemia and hypovolemic shock in pregnant women. Hence, it is helpful for obstetricians and anesthesiologists to prepare pre-emptive prevention when predicting PPH occurrence in advance. However, current works on PPH prediction focus on whether PPH occurs rather than assessing PPH amount. To this end, this work studies quantitative PPH prediction with machine learning (ML).

**Methods:**

The study cohort in this paper was selected from individuals with PPH who were hospitalized at Shijiazhuang Obstetrics and Gynecology Hospital from 2020 to 2022. In this study cohort, we built a dataset with 6,144 subjects covering clinical parameters, anesthesia operation records, laboratory examination results, and other information in the electronic medical record system. Based on our built dataset, we exploit six different ML models, including logistic regression, linear regression, gradient boosting, XGBoost, multilayer perceptron, and random forest, to automatically predict the amount of bleeding during cesarean section. Eighty percent of the dataset was used as model training, and 20$\%$ was used for verification. Those ML models are constantly verified and improved by root mean squared error(RMSE) and mean absolute error(MAE). Moreover, we also leverage the importance of permutation and partial dependence plot (PDP) to discuss their feasibility.

**Result:**

The experiment results show that random forest obtains the highest accuracy for PPH amount prediction compared to other ML methods. Random forest reaches the mean absolute error of 21.7, less than 5.4$\%$ prediction error. It also gains the root mean squared error of 33.75, less than 9.3$\%$ prediction error. On the other hand, the experimental results also disclose indicators that contributed most to PPH prediction, including Ca, hemoglobin, white blood cells, platelets, Na, and K.

**Conclusion:**

It effectively predicts the amount of PPH during a cesarean section by ML methods, especially random forest. With the above insight, ML predicting PPH amounts provides early warning for clinicians, thus reducing complications and improving cesarean sections’ safety. Furthermore, the importance of ML and permutation, complemented by incorporating PDP, promises to provide clinicians with a transparent indication of individual risk prediction.

## Introduction

Cesarean section hemorrhage is one of the common complications in cesarean section [1]. Blood loss exceeding 1000 milliliters within the first 24 hours after the cesarean section is qualified as postpartum hemorrhage (PPH). PPH is a significant global health concern [2]. Severe hemorrhage caused by cesarean section potentially brings serious consequences, even maybe maternal death [3]. Hence, it is significant to accurately predict the amount of PPH during cesarean section and take early preventive measures to improve the delivery safety of pregnant women [4]. Currently, clinicians mainly rely on clinical experience to use statistical models to predict the occurrence of PPH. It heavily relies on healthcare professionals’ clinical practice and statistical abilities [5].

Statistical prediction methods are not conducive to situations with a shortage of professional clinicians [6]. There is potential to enhance predictive accuracy by applying machine learning (ML) methods [7]. Compared to traditional statistical models, ML has gained significant attention due to its superior predictive capabilities [8]. ML offers advantages such as processing non-additive relationships and incorporating complex interactions between indicators without needing pre-specification [9]. The ML model has been widely used in PPH prediction of cesarean section. These models can be trained through large-scale clinical data and output corresponding hemorrhage predictions based on input predictive indicators [10]. The model mainly uses mainstream ML methods, such as support vector machine (SVM), random forest, and artificial neural network (ANN) to predict PPH.

However, current works about PPH prediction mainly focus on constructing classification models [11]. Because PPH accounts for no more than 8$\%$ of all pregnant women, the research data is highly imbalanced. Even the final classification accuracy of the classification model is high. But the missed detection rate is also high, causing a low recall rate [8].

Comparing predicting whether PPH occurs, the advantages of quantitative prediction of PPH are apparent. First, quantitative prediction of PPH can intuitively predict the specific amount of bleeding. It provides convenience for clinicians to conduct preoperative evaluations. Second, it is conducive to establishing a graded diagnosis and treatment system. Clinicians can transfer critically sick pregnant women to advanced hospitals in advance to avoid the waste of cutting-edge medical resources. Third, It is beneficial for clinicians to allocate blood transfusion resources reasonably. At present, a blood transfusion unit is a bag of 100 milliliters. It needs to be thawed one hour in advance. The PPH regression model could reasonably arrange blood transfusion volume and thawing time [12].

Therefore, studying and evaluating the amount of cesarean section hemorrhage is significant. This study aims to identify and assess predictive indicators related to bleeding volume and establish reliable quantitative prediction models. The quantitative prediction models can help clinicians accurately determine the risk of hemorrhage during cesarean section and take treatment measures [13]. There are three contributions to the quantitative prediction of PPH in this study:

- **A self-organized dataset.** The study cohort in this research was drawn from individuals hospitalized at Shijiazhuang Obstetrics and Gynecology Hospital from 2020 to 2022. Within this study cohort, a dataset comprising 6,144 subjects was constructed. This dataset encompassed an array of clinical parameters, anesthesia operation records, laboratory examination results, and other pertinent information extracted from the electronic medical record system.
- **Verification and comparison of various ML methods.** Utilizing the dataset we constructed, we employ six distinct ML models. The ML models comprise logistic regression, linear regression, gradient boosting, XGBoost, multilayer perceptron, and random forest. The ML models achieve the prediction of hemorrhage volume during cesarean sections. These ML models receive verification and refinement through a self-learning mechanism. Additionally, we employ permutation importance and partial dependence plot (PDP) to evaluate their feasibility.
- **Important indicators discovery.** We discover the most risk indicators for the quantitative prediction of PPH. It relies on reasonable data processing and comparing different ML models. The importance of risk indicators is sorted.

## Related works

### Indicator exploration

Many researchers are striving to identify risk indicators highly associated with PPH. Kumar et al. [14] designed an automated method using wearable devices to prevent PPH in pregnant women. The wearable devices assess parameters including perspiration rate, temperature, pulse rate, and blood pressure. The method incorporates fuzzy neural-based rules for each parameter to predict the risk of PPH. The accuracy of this method is evaluated by decreasing morbidity and mortality rates associated with PPH. The type of emergency cesarean section is related to whether the uterine incision is longitudinal or transverse [15]. Wu et al. [16] endeavored to construct a nomogram that integrates both clinical and radiomic features of the placenta to forecast the risk of PPH occurring in a cesarean section. Radiomic features are selected based on their correlation with PPH. Various methods, including clinico-radiomic, radiomic, radiological, clinical, and clinico-radiological approaches, are developed for predicting PPH risks in all individuals. The method with superior predictive accuracy is validated through the clinical application, discrimination ability assessment, and calibration curve analysis.

Krishnamoorthy et al. [17] presented an approach for predicting PPH by introducing the oppositional binary crow search algorithm (OBCSA) coupled with an optimal stacked autoencoder (OSAE) model, denoted as OBCSA-OSAE. This technique encompasses OBCSA-based feature selection methods strategically employed to determine an optimal subset of features. They found that influencing preoperative indicators include maternal age, weight, gestational age, pregnancy complications (such as preeclampsia, placental abruption, etc.), and abnormal coagulation function. Heesen et al. [18] found out indicators such as older pregnant women, earlier gestational weeks, and the presence of preeclampsia were associated with a higher rate of PPH. It is necessary to build a reasonable dataset and identify the indicators strongly correlated with PPH. It is the key to comprehensively understanding the indicators influencing these predictions.

### Prediction tasks

Many studies have identified multiple predictive indicators associated with the occurrence of cesarean section hemorrhage, primarily focusing on the prediction of classification of whether PPH occurs or not. Those studies try to find the indicators that have a strong correlation with PPH rather than the prediction of the volume of PPH [19]. These indicators can be divided into preoperative, intraoperative, and postoperative indicators. Betts et al. [10] aimed to predict the risks of general maternal postpartum complications in inpatient care. They employed a gradient boosting tree with 5-fold cross-validation to compare method accuracy. The methods demonstrating superior performance for all outcomes were subsequently evaluated in independent data. The methods were validated by the area under curve (AUC) and receiver operating characteristic (ROC) method.

Zhong et al. [20] analyzed to identify risk indicators associated with various degrees of PPH in patients with pregnancy-induced hypertension. They applied a line graph to construct the predictive model. The study revealed that the severity of the disease, gestational week upon onset, gestational week upon delivery, degree of proteinuria, systolic blood pressure, diastolic blood pressure, and uterine atony are significant indicators. The indicators influence the incidence of PPH in patients with hypertensive disorder complicating pregnancy. The resulting prediction model, based on these indicators, demonstrates accurate capabilities in assessing the risk of diverse degrees of PPH in patients with hypertensive disorders complicating pregnancy. The datasets in the studies exhibit limited sample sizes, rendering them less representative of the broader population for quantitative prediction. Consequently, even if a model is constructed with high classification accuracy, its practical utility for real-world quantitative prediction research is diminished.

### Model selection

ML has been increasingly integrated into scientific discovery to accelerate research in recent years. The application of ML methods for developing PPH classification prediction models has become increasingly prevalent. Venkatesh et al. [15] employed logistic regression with and without lasso regularization, constituting two distinct statistical approaches. Additionally, they utilized XGBoost and random forest as the two ML methods for the prediction of PPH. Model accuracy assessment involved using calibration, decision curves, and C-statistics. The results show that the XGBoost model provided the most significant net benefit. Liu et al. [21] created a dataset comprising 850 cases of PPH and applied various ML models to enhance the precision of predicting PPH in vaginal delivery. The study compared the accuracy of predictions among an assessment table, classical statistical models, and ML models to assess their clinical utility. The assessment table featured 16 key risk indicators for PPH prediction. The classical statistical model employed was logistic regression. The ML models included random forest, k-nearest neighbor (KNN), and a hybrid model integrating light gum and logistic regression. Model performance was evaluated through metrics such as AUC, specifically C-statistic, calibration curve brier score, decision curve, F-measure, sensitivity, and specificity. Among the evaluated models, the ML model light gum + logistic regression demonstrated superior performance in predicting PPH.

Paydar et al. [22] implemented a univariate logistic regression method to select important features (P<0.01). Subsequently, they employed multiple ANN and binary logistic regression methods to predict PPH, encompassing radial basis function (RBF), multilayer perceptron, and backpropagation in the neural network methods. The identification and comparison of precise networks were conducted using the ROC curve and the confusion matrix. There is scant research on the quantitative prediction of PPH. The absence of interpretability and intuitive comprehension in ML models is a significant impediment to PPH research.

## Methodology

**Fig. 1:**
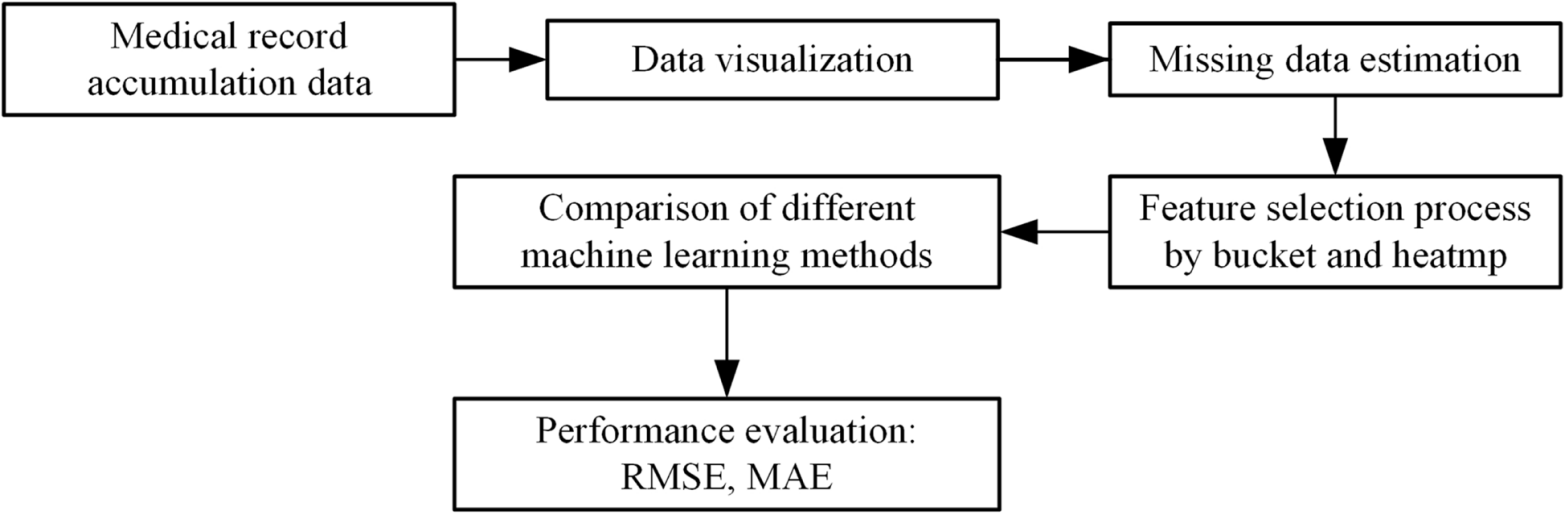
Flow chart

We aimed to develop and validate quantitative prediction models for PPH using data processing and ML method selection. We analyzed preoperative and intraoperative data in cesarean section. We comprehensively analyzed the correlation between these data and the prediction of bleeding volume. We compared the ability of various ML and deep learning models to utilize these predictive data. Finally, a random forest regression method is applied to predict the amount of cesarean section PPH effectively. This study used preoperative and intraoperative examination data for preprocessing and selected reasonable and adequate indicators. The indicators were applied to the appropriate ML methods to predict bleeding volume. It is helpful for the following work, such as analyzing effective indicators in the prediction model and providing references for clinicians. Figure 1 shows the main research steps.

### Dataset acquisition

The data in this study were selected from delivery women hospitalized at the Shijiazhuang Obstetrics and Gynecology Hospital from 2020 to 2022. The hospital has an average annual delivery volume of 28,000. There are a total of 6,144 pieces of individuals in this dataset. A total of 54 indicators were collected per patient. According to previous studies [14, 17–19], we selected 27 indicators that were potentially clinically related to PPH. All data comes from the medical electronic case system. In terms of the maternal bleeding situation, there is a class imbalance in the dataset. This issue is solved by establishing feature engineering when using different ML methods for training. The study adopts a retrospective analysis method [23]. Based on the guidelines for the management and prevention of PPH issued by obstetrics and gynecology, we use both volumetric and gravimetric methods to calculate blood loss. We conduct regular training sessions for evaluators. First, we calculate blood loss using the volumetric method. During a cesarean section, an assistant uses a vacuum suction device to collect as much amniotic fluid as possible after the amniotic sac is ruptured. The assistant records the amount of amniotic fluid in the suction device. The volumetric blood loss is equal to the volume in the suction device minus the amount of amniotic fluid and irrigation fluid. Then, we calculate the remaining blood loss on the operating table using the gravimetric method. The weight of surgical drapes and perineal pads is calculated before and after the surgery. Gravimetric blood loss (ml) = (post-surgery pad weight - pre-surgery pad weight) / 1.05. The total blood loss is the sum of the blood loss calculated by the volumetric and gravimetric methods.

We extract subject information from the hospital medical record information system. The indicators include fundamental information about pregnant women, such as age, height, weight, operation diagnosis, number of pregnancies (NOP), gestational week, complications, blood pressure, and infant weight (IW). The indicators include hematological indices such as hemoglobin (HB), white blood cell (WBC), and platelet (PLT). The indicators include coagulation function indicators such as prothrombin time (PT), international standardized ratio (INR), activated partial thromboplastin time (APTT), thrombin time (TT), and fibrinogen quantification (FIB). The indicators include liver function tests, such as bilirubin, ALT, and AST. The indicators include renal function tests such as urea and creatinine. The indicators include ion examination such as Na, K, Cl, and Ca. The indicators also include surgical indicators such as anesthesia method, ASA, and emergency treatment (ET). The selection of these indicators is mainly based on previous research and the professional experience of clinicians. In the dataset, HB, WBC, PLT, PT, INR, APTT, TT, FIB, Na, K, Cl, Ca, bilirubin, urea, creatinine, weight, height, IW, age, NOP, gestational week, blood pressure, complication, PPH are continuous data. The anesthesia method, ASA, ET, and complications are categorical data.

### Exploratory data analysis

**Fig. 2:**
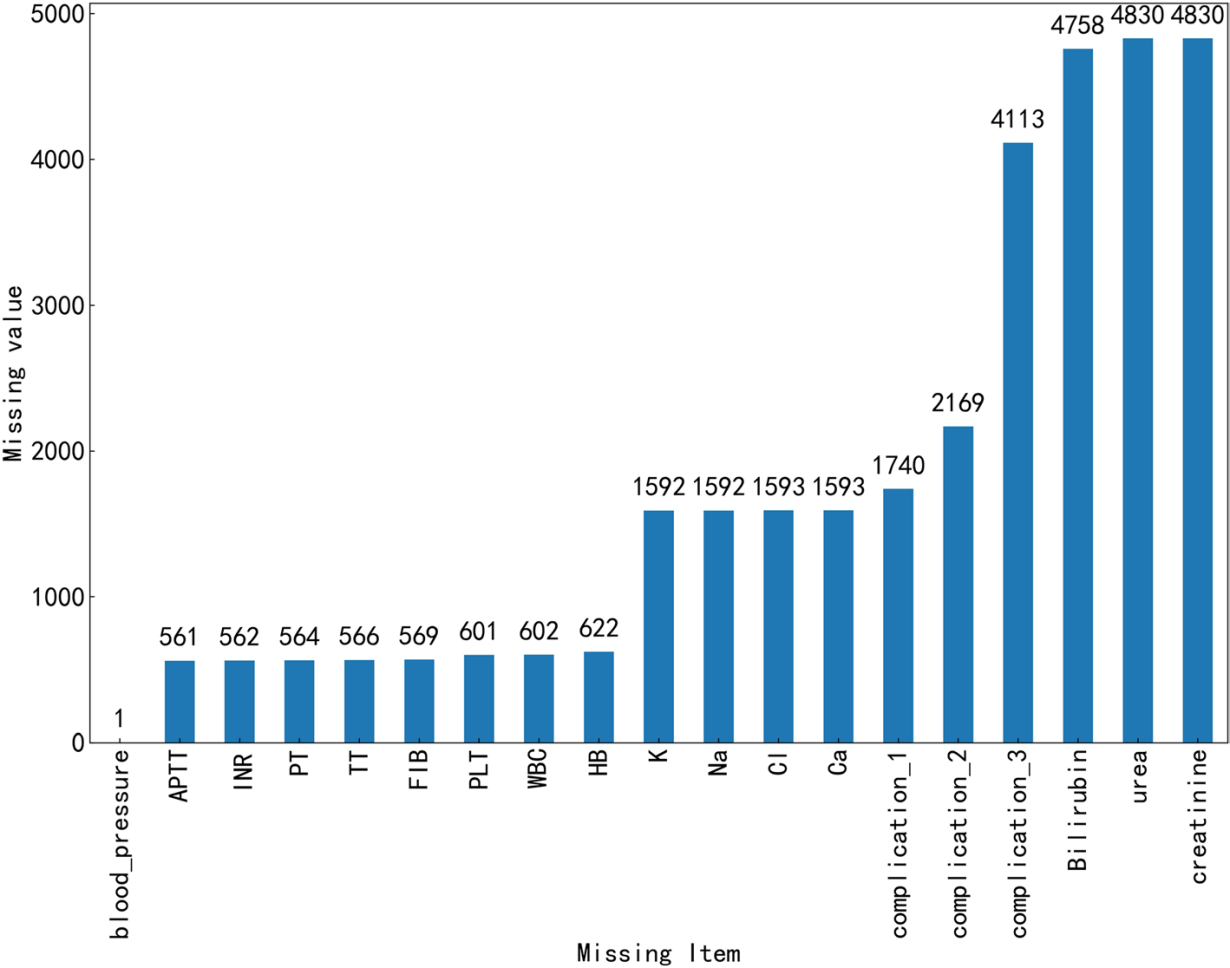
Missing data analysis

Exploratory data analysis (EDA) is a critical task in predicting PPH. In this paper, we conducted an EDA on the amount of hemorrhage and related research indicators, including the patient’s physical examination, data distribution, missing data, outliers, etc. EDA helps us explore the characteristics of hemorrhage volume data, identify data quality issues, and prepare for further data processing and analysis [24]. Meanwhile, the data we obtained is not complete. The obtained data contains specific missing data. A certain proportion of missing data exists due to the negligence of recording personnel. The missing data analysis in this paper is shown in Fig. 2. It is also helpful for the following model development.

There is a small number of missing data in APTT, INR, PT, TT, FIB, PLT, WBC, HB, K, Na, Cl, Ca, etc. It is due to the loss of clinical personnel records. Complication_1, complication_2, and complication_3 belong to the category of prenatal complications. Only some pregnant women have one or several related diseases. So, a large proportion of missing data is expected. Bilirubin, urea, and creatinine have high miss rates. The reason is that many pregnant women have not undergone this examination before cesarean section. It requires reasonable processing in the following part of data processing.

**Fig. 3:**
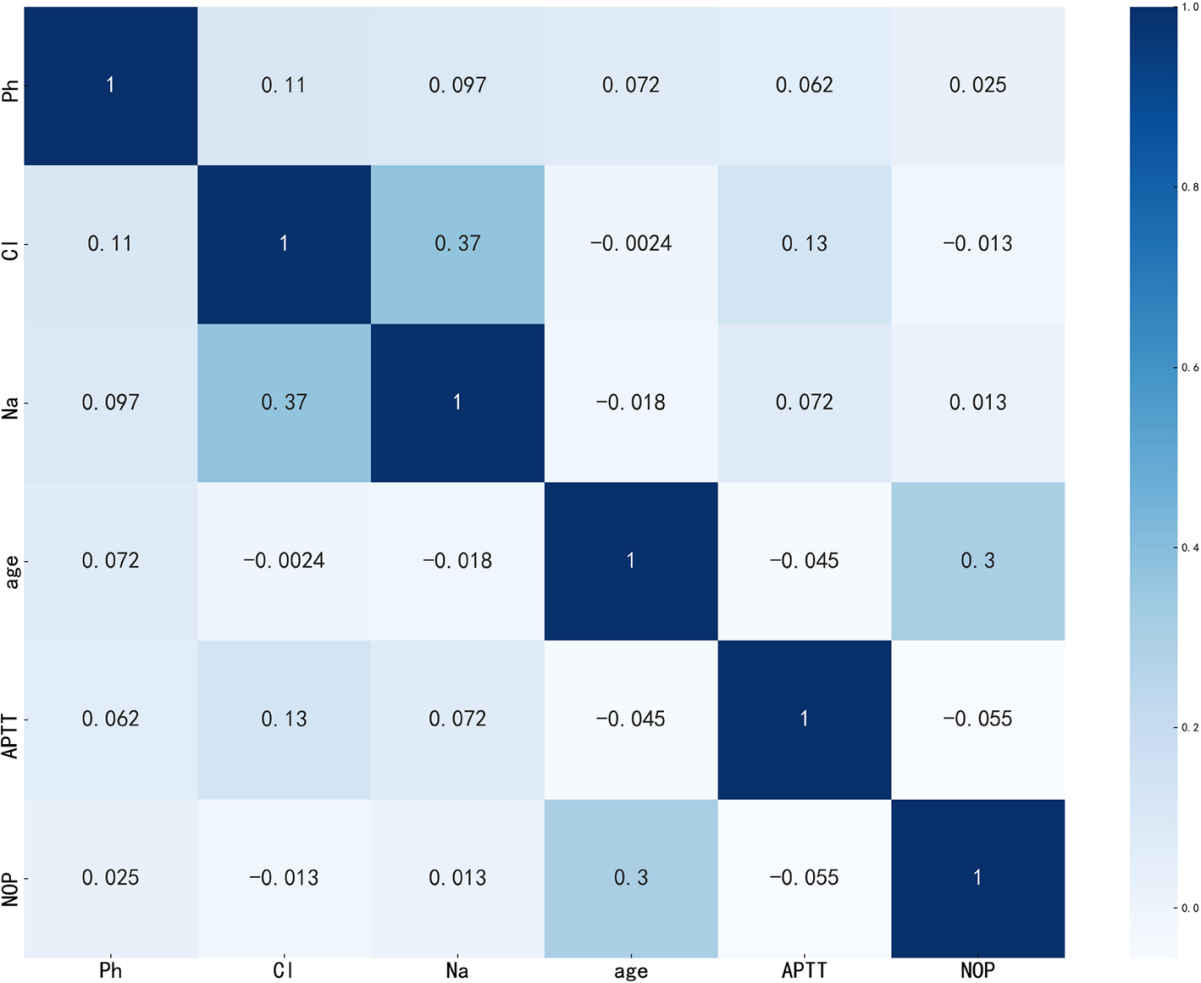
Correlation analysis of some indicators

The next step is to conduct a correlation analysis of the data. Correlation analysis can help researchers reveal the relationships between indicators [25]. This paper determines the strength of the correlation between indicators by calculating the Pearson correlation coefficient. Analysis of the correlation between different indicators of hemorrhage helps researchers reveal patterns, trends, and dependencies in the data. It provides a theoretical basis for subsequent modeling and prediction [26]. We explored which indicators have a strong correlation with maternal hemorrhage. The correlation of the indicators is valuable for feature selection. Because it can help researchers eliminate features that have a weak relationship with the target indicator, it helps to improve the effectiveness and efficiency of modeling. We further explore multivariate relationships. This study explores the overall correlation pattern between multiple indicators by calculating the correlation coefficients between hemorrhage volume and other continuous indicators. This study discovers complex interactions between indicators and analyzes some indicators strongly correlated with hemorrhage. The correlation matrix obtained is shown in Fig. 3.

**Fig. 4:**
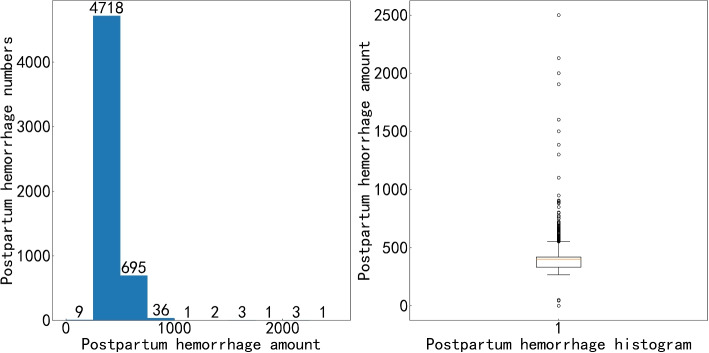
Hemorrhage bar chart and box chart

This work provides a foundation for the follow-up feature engineering through the hemorrhage EDA. By observing and analyzing the indicators of the dataset, essential indicators related to the target indicator can be preliminarily identified. Some indicators could be transformed, combined, or derived. In this paper, we made a bar chart and a box chart to show the distribution of hemorrhage. We found that there was uneven distribution in the dataset. According to conventional operating methods, researchers maintain the original distribution pattern for subsequent prediction modeling. Considering the possibility of massive hemorrhage, outliers were not processed in this paper. We maintain the actual distribution pattern for the following prediction model. The data distribution analysis is shown in Fig. 4. We could see that PPH occurs at low frequency. The study on the accurate amount of PPH is meaningful.

### Data preprocessing

**Mean and mode imputation** Two primary strategies for mitigating the challenges posed by missing data are deletion and imputation. Deletion methods involve ignoring missing data and are straightforward procedures that rely on fully recorded samples, as exemplified by Zhou et al. [27]. While deletion is convenient, caution should be exercised in its application, as it can potentially introduce bias into the analysis [28]. Regarding imputation methods, their primary purpose is to substitute or replace missing data with predicted values, typically estimated from the observed data. Predominant techniques encompassed within statistical and ML frameworks involve mean imputation, regression imputation, stochastic regression imputation, hot-deck imputation, and KNN imputation.

- **Mean imputation** is a principal approach to handling missing data. It involves replacing missing data with the arithmetic mean of observed data points. The rationale behind mean imputation lies in its capacity to preserve the overall distribution of the indicators. It is particularly effective when confronted with symmetrically distributed indicators.
- **Mode imputation** is germane to categorical indicators. It involves replacing missing data with the mode and representing the most frequently occurring category. The application of mode imputation is apt when particular categories substantially dominate the distribution. It is particularly effective when the categorical nature of the indicator precludes the use of mean or median imputation.

In this study, the missing data significantly impacted subsequent ML modeling work. Considering that there are continuous and categorical indicators in the dataset, the processing method in this study uses the mean value to fill in the continuous indicators and the mode value to fill in the categorical indicators. This processed method can maintain the overall distribution and mean of the data unchanged. The advantage of completing by the mean value is that it is simple and easy to implement without introducing new deviations. This paper fills in indicators including APTT, INR, PT, TT, FIB, PLT, WBC, HB, K, Na, Cl, and Ca with mean values. The missing rate for the three indicators, including bilirubin, urea, and creatinine, is over 75$\%$. Because the missing rate is too high, we delete the indicators directly and discard the dirty data. We split blood pressure values into diastolic blood pressure (DBP) and systolic blood pressure (SBP).

For the missing categorical indicators in this study, such as ASA, the mode value can be calculated by the non-missing data. We fill in the missing data by the mode value for the categorical indicators. Mode imputation can maintain the distribution characteristics and relative proportion of data without introducing new categories. Both methods are effective interpolation methods and are suitable for dealing with situations where missing data are randomly distributed in this study.

**The bucket method**

Discretization plays a crucial role in preparing data for predictive modeling. It requires transforming continuous or categorical indicators into categorical representations. The bucket method involves partitioning the range of a categorical indicator into distinct intervals. Each distinct interval represents a categorical entity. This study applies the bucket method to convert numeric representations into categorical representations, facilitating model interpretation and potentially capturing nonlinear relationships. The bucket method is used to process categorical indicators in the dataset to make them more effective in the subsequent model prediction process. The primary operating process is as follows:

- We divide the prenatal symptoms of complications into pregnancy with hypothyroidism (PWH), pregnancy thrombocytopenia (PT), preeclampsia, placental abruption (PAB), gestational diabetes (GD), intrapartum fever (IF), pregnancy associated with hysteromyoma (PAH), chorioamnionitis, pregnancy-induced hypertension (PIH), placenta, placenta accreta (PAC), other prenatal symptoms (OPS), no prenatal symptoms (NPS).
- We divide anesthesia methods into combined spinal-epidural anesthesia (CSEA), epidural block anesthesia (EBA), general anesthesia (GA), spinal anesthesia (SA), and other anesthesia methods (OAM).
- We divide anesthesia level into ASA_L1, ASA_L2, ASA_L3, ASA_L4, ASA_L5.
- We divide emergency treatment into ET_emergency, ET_predict.
- We use a natural language processing method to record the parity of pregnant women from pregnancy detection records.
- We create an indicator of whether the pregnant woman has a twin by 1 and 0.

For missing data in categorical indicators, the mode imputation is applied. Table 1 shows the final selected prediction indicators.

**Table 1:** Prediction indicators

Index	Column	Index	Column	Index	Column
1	HB	17	NOP	33	Parity
2	WBC	18	DBP	34	Twins
3	PLT	19	SBP	35	OAM
4	PT	20	PWH	36	CSEA
5	INR	21	POD	37	EBA
6	APTT	22	Preeclampsia	38	GA
7	TT	23	PAB	39	SA
8	FIB	24	GD	40	ASA_L1
9	Na	25	IF	41	ASA_L2
10	K	26	PAH	42	ASA_L3
11	Cl	27	Chorioamnionitis	43	ASA_L4
12	Ca	28	PIH	44	ASA_L5
13	Weight	29	Placenta	45	ET_emergency
14	Height	30	PAC	46	ET_predict
15	IW	31	OPS	47	Pregnancy_Days
16	Age	32	NPS		

After completing data cleaning and processing, 5,468 pieces of experimental data can be used for various ML models for research. Three pieces of the processed data in the study were shown in Table 2. We presented some processed data for a more intuitive understanding of data generation. It can be seen that the missing continuous and categorical indicators were reasonably processed by mean and mode imputation. Employing the bucket method, we transformed categorical data into a data form that the ML model can process. It facilitates the next step of building a PPH quantitative prediction model.

**Table 2:** Processed data preview

ID	1	2	3	ID	1	2	3
HB	117	117	97	GD	0	0	0
WBC	11.89	11.89	10.6	IF	0	0	0
PLT	383	383	200	PAH	0	0	0
PT	11.4	11.4	11.7	Chorioamnionitis	0	0	0
INR	0.95	0.95	0.98	PIH	0	0	0
APTT	32.3	32.3	36.1	Placenta	0	0	0
TT	14.3	14.3	14.1	PAC	0	0	0
FIB	3.6	3.6	4.24	OPS	1	1	1
Na	137.5	137.5	137	NPS	0	0	0
K	3.98	3.98	3.88	Parity	1	1	1
Cl	102.5	102.5	101.6	Twins	1	1	0
Ca	2.15	2.15	1.99	OAM	0	0	0
Ph	573	573	547	CSEA	1	1	0
Weight	62	62	70	EBA	0	0	1
Height	160	160	158	GA	0	0	0
IW	1530	990	3530	SA	0	0	0
Age	29	29	27	ASA_L1	0	0	0
NOP	1	1	1	ASA_L2	1	1	1
DBP	80	80	78	ASA_L3	0	0	0
SBP	128	128	122	ASA_L4	0	0	0
PWH	1	1	0	ASA_L5	0	0	0
POD	0	0	0	ET_emergency	0	0	1
Preeclampsia	0	0	0	ET_predict	1	1	0
PAB	0	0	0	Pregnancy_Days	233	233	276

### Model development

To quantitatively predict PPH, we utilized six ML models on the processed dataset. They are logistic regression, linear regression, gradient boosting, XGBoost, multilayer perceptron, and random forest.

- **Logistic regression.** Logistic regression algorithm is a statistical model used for probabilistic nonlinear regression, estimating the probability of an event occurrence based on input indicators. It is particularly suited for problems where the outcome is categorical.
- **Linear regression.** Linear regression is a foundational statistical technique for predicting a continuous outcome. It needs to model the linear relationship between the dependent indicators.
- **Gradient boosting.** Gradient boosting is an ensemble ML method that builds a predictive model incrementally. It minimizes error in prediction by combining weak learners.
- **XGBoost.** XGBoost is an optimized implementation of gradient boosting, recognized for its efficiency and performance in predictive modeling. It excels in both regression and classification tasks, often outperforming other algorithms.
- **Multilayer perceptron.** Multilayer perceptron is a type of ANN with multiple layers of interconnected nodes. It can capture complex patterns and relationships within a dataset, making it suitable for intricate regression tasks.
- **Random forest.** Random forest is an ensemble learning algorithm that constructs multiple decision trees during training. It aggregates their predictions to enhance accuracy in regression tasks, which is particularly beneficial for handling noisy data and avoiding overfitting.

## Experiments

### Evaluation metrics

After completing the data processing, we compared different ML models for analysis. When selecting an ML regression model in this article, the following criteria are comprehensively considered:

- The model’s performance is the primary criterion for selecting the model. This study applies several common evaluation indicators to measure the performance of the hemorrhage prediction model, including root mean squared error(RMSE) and mean absolute error(MAE). Both of them are defined in Equation 1 and 2, respectively. The lower error value indicates that the model’s prediction is more accurate.
  1 $$\begin{aligned} RMSE = \sqrt{\frac{1}{n}\sum \limits _{i = 1}^n ({y_i} -{\hat{y_i}})^2} \end{aligned}$$
  2 $$\begin{aligned} MAE = \frac{1}{n}\sum \limits _{i = 1}^n {|{y_i} - {\hat{y_i}}|} \end{aligned}$$
- The interpretability of the model is another criterion for selecting the model. As a medical research project, the interpretability of hemorrhage prediction models is significant, especially when it is necessary to understand the relationships between indicators [29]. The ML model used in this study has good interpretability and can help explain the influence relationships between indicators [30]. This paper uses PDP to illustrate the marginal impact of the given indicators on the experiment result to show interpretability.

Considering the above Criteria comprehensively, we analyze the generalization abilities of ML models and the characteristics of the data set. This paper selects random forest regression as an optimal algorithm. Random forest regression is adaptable to high-dimensional datasets with many indicators and can select the most critical indicators from the dataset. Because the random forest comprises multiple decision trees, each tree can capture different nonlinear patterns in the data. So, the random forest model can deal with the nonlinear relationship between characteristic indicators and prediction values.

### Experimental setup

This work is based on Python 3.8 and utilizes Sklearn’s ML toolkit to complete the modeling work. We apply the grid search method to decide the best parameters for the random forest regression. The grid search method’s search range of random forest is [10,500]. The search scope for internal nodes is (2,5,10). The parameters of the leaf node are set to (1,2,4,8). We divide the collected hemorrhage data into training and testing sets in an 8:2 ratio. The parameters obtained by grid search are n_estimators: 1250, min_samples_split: 2, min_samples_leaf: 1, max_features: sqrt, max_depth: 90. This study suggests trying multiple models and comparing their performance.

### Experimental results

#### Quantitative performances

This paper selects logistic regression, linear regression, gradient boosting, XGBoost, multilayer perception (The hidden laters size is 20,30,10), and random forest regression for regression analysis. The average amount of postpartum bleeding is 397 milliliters. The MAE of the random forest regression prediction model is 21.7 milliliters, which is less than the 5.4$\%$ prediction error. The RMSE of the random forest regression prediction model is 33.75 milliliters, which is less than the 9.3$\%$ prediction error. The predicted results are shown in Table 3. The results show that random forest regressor have better performance than other methods.

**Table 3:** Comparasion for different methods on the in-house dataset

Model	MAE	RMSE
Multilayer Perceptron	63.35	101.65
Logistic Regression	59.43	104.16
Linear Regression	59.11	88.45
Gradient Boosting	53.97	73.23
XGBoost	23.67	34.69
**Random Forest**	**21.70**	**33.75**

**Fig. 5:**
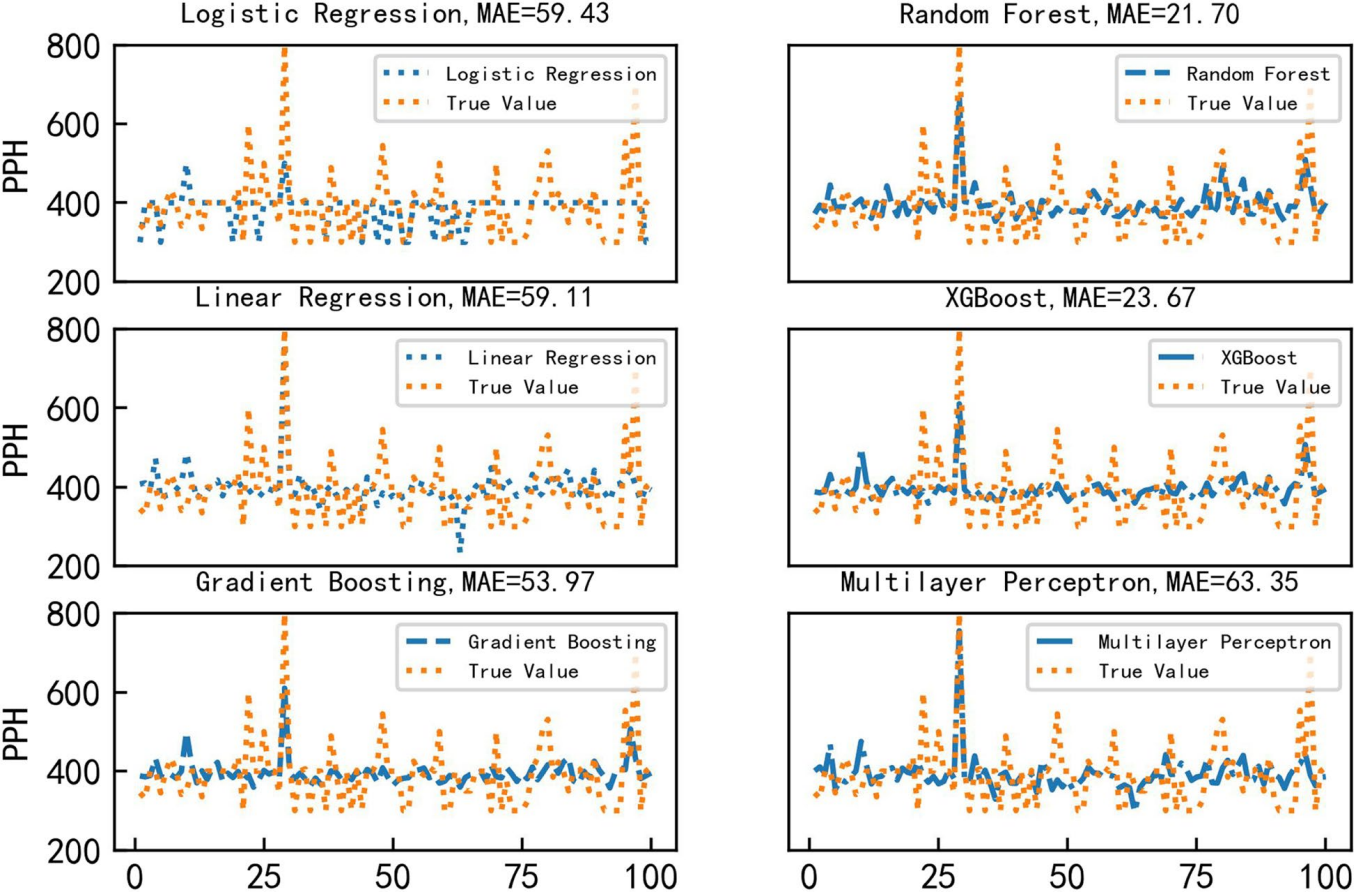
Regression analysis results of several ML methods

Multiple ML prediction models were applied to the test set for analysis, and the fitting results obtained are shown in Fig. 5. We could see that random forest performs better than other ML methods. Its MAE is 1.97 less than that of the second-best-performing model, XBGoost. Its RMSE is 0.94 less than that of the second-best-performing model, XBGoost. The multilayer perceptron is the worst-performing model. From the perspective of model principles, random forest is an ensemble learning method that combines the predictions of multiple decision trees. The superior performance of random forest in PPH prediction can be attributed to its ensemble nature, robust handling of nonlinear relationships, and efficient processing of large datasets. XGBoost follows closely, leveraging its computational efficiency and generalization capabilities. On the other hand, XGBoost performs well as it is an advanced implementation of gradient boosting and is known for its efficiency, scalability, and high predictive accuracy. In contrast, the multilayer perceptron may perform comparatively worse because it is sensitive to the scale and distribution of input features. It requires extensive tuning of hyperparameters and is susceptible to overfitting, especially when dealing with small datasets like those in PPH prediction.

#### Permutation importance

**Fig. 6:**
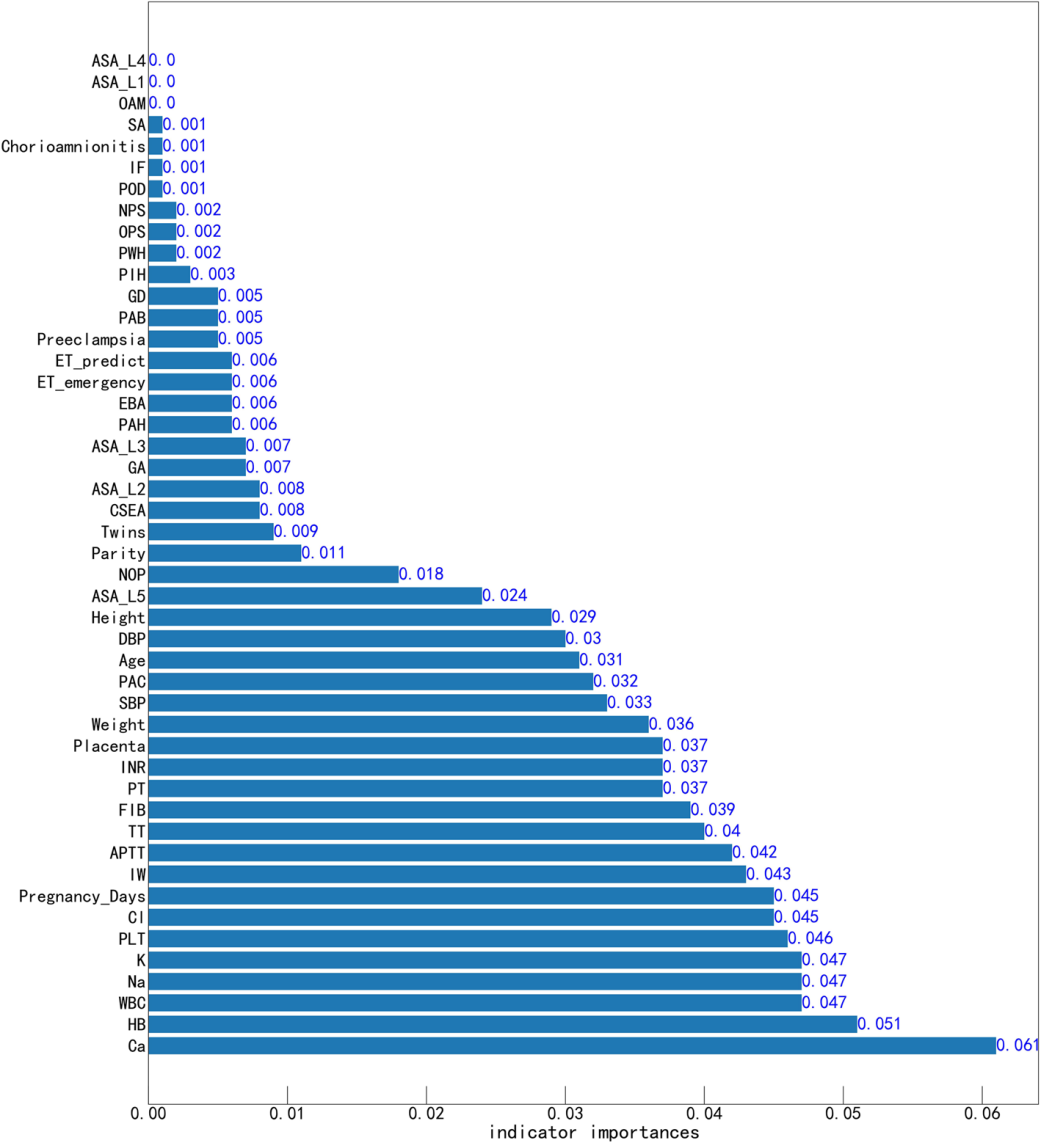
Permutation importance of the indicators

Permutation importance constitutes an initial tool for comprehending ML models. It serves as a valuable technique for elucidating the predictive potential of each indicator within these models. This method involves systematically altering individual indicators in the validation dataset and observing resultant changes in accuracy. The significance of each indicator is established through ranking, with the top value denoting the most influential, while the bottom value signifies relatively lesser importance. Through the indicator importance analysis of the random forest with the best prediction effect, Ca, HB, WBC, PLT, Na, and K are the top six indicators contributing to the prediction model. The histogram analysis of the importance of indicators is shown in Fig. 6.

Specifically, electrolyte detection, such as Ca, Na, and K ion content, is critical in the predictive modeling of bleeding volume. These indicators contribute to hemorrhagic events. These ions play essential roles in various physiological processes, and their contents can potentially affect blood coagulation, vessel integrity, and other hemostatic mechanisms. Investigating the relationship between the concentrations of Ca, Na, and K ions and the prediction of bleeding volume is integral for gaining insights into the underlying physiological mechanisms. It enhances the accuracy of predictive models. Ca, in particular, is a critical cofactor in the blood coagulation cascade. It activates various clotting factors that are necessary for forming a stable blood clot [31]. Low calcium levels can impair clot formation, leading to increased bleeding risks during and after cesarean sections. Na plays a vital role in maintaining fluid balance and blood pressure [32]. Hyponatremia can lead to hemodilution, affecting coagulation and increasing bleeding risks. K is crucial for cellular function and maintaining the electrical conductivity of cells [33]. Abnormal potassium levels can affect muscle contractions, including those of the uterine muscles, potentially leading to increased bleeding during childbirth.

Furthermore, blood examinations for HB, PLT, and WBC are essential in predicting PPH. HB is crucial for oxygen transport and indicates the blood’s oxygen-carrying capacity. Low hemoglobin levels, known as anemia, are associated with an increased risk of bleeding during and after childbirth. Adequate hemoglobin levels are essential for maintaining hemostasis and preventing excessive bleeding [34]. HB is crucial for oxygen transport and indicates the blood’s oxygen-carrying capacity. Low HB levels, known as anemia, may result in increased bleeding risk during and after childbirth [35]. PLTs are critical components in blood clotting. Adequate platelet levels are necessary for the formation and stability of blood clots. Low platelet counts, or thrombocytopenia, can impair clotting and contribute to PPH. WBCs are integral to the immune system and inflammation response. Elevated or decreased white blood cell counts may indicate underlying infections or inflammatory conditions that could impact the body’s ability to manage postpartum bleeding. Monitoring WBC counts can help identify and manage potential complications that could exacerbate bleeding risks [36].

#### Partial dependence plot

**Fig. 7:**
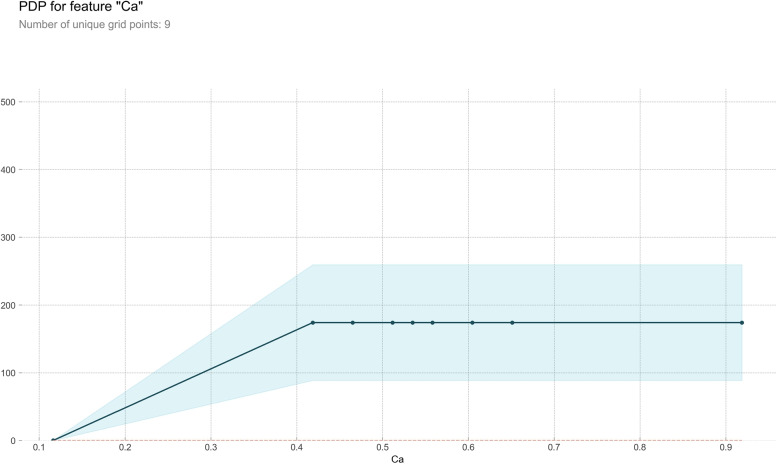
The PDP of indicator Ca

**Fig. 8:**
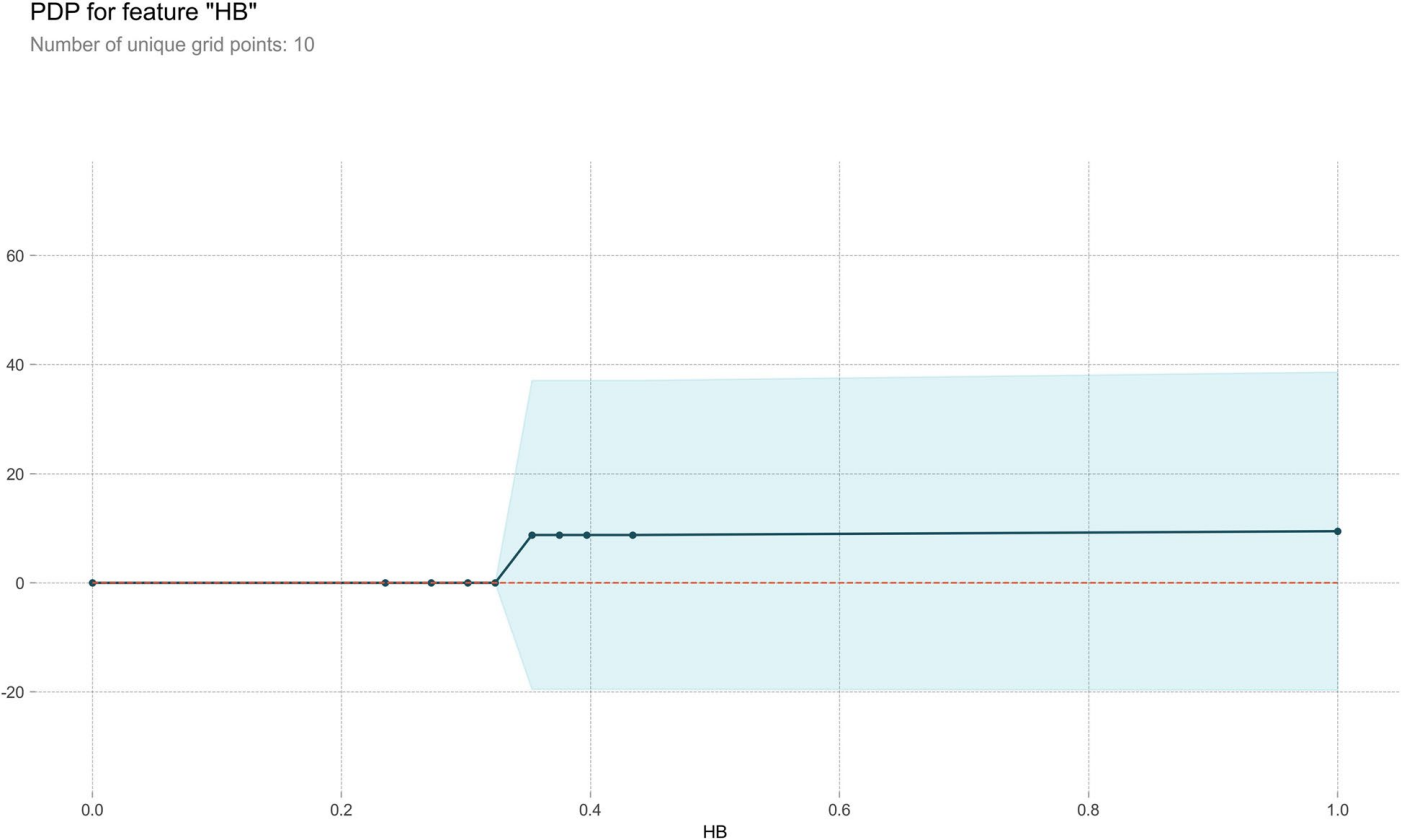
The PDP of indicator HB

**Fig. 9:**
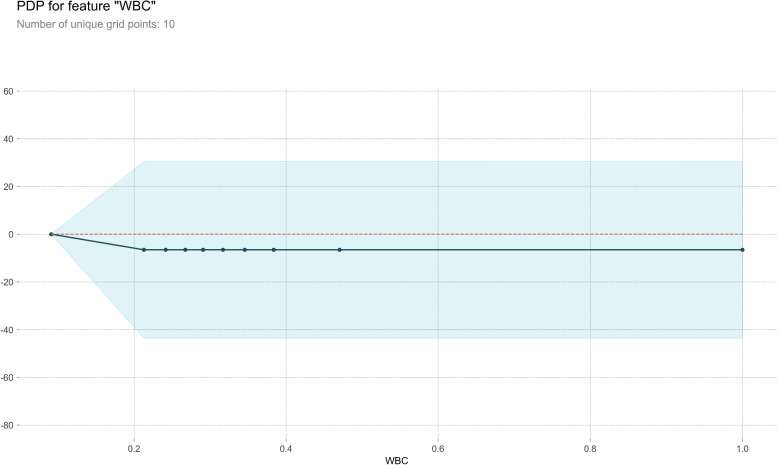
The PDP of indicator WBC

**Fig. 10:**
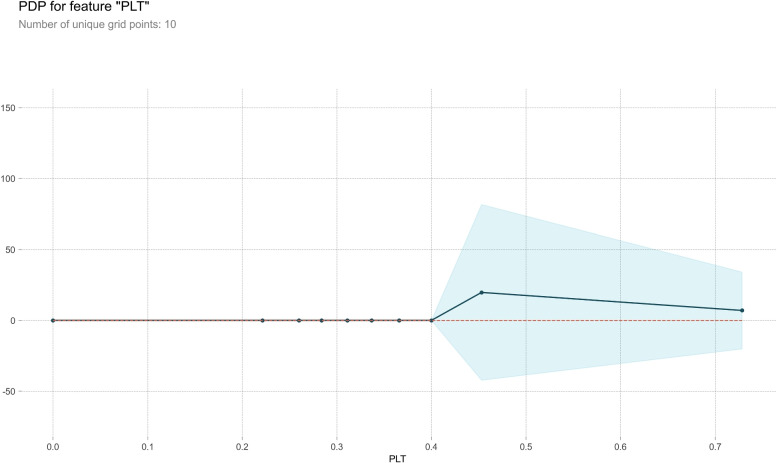
The PDP of indicator PLT

**Fig. 11:**
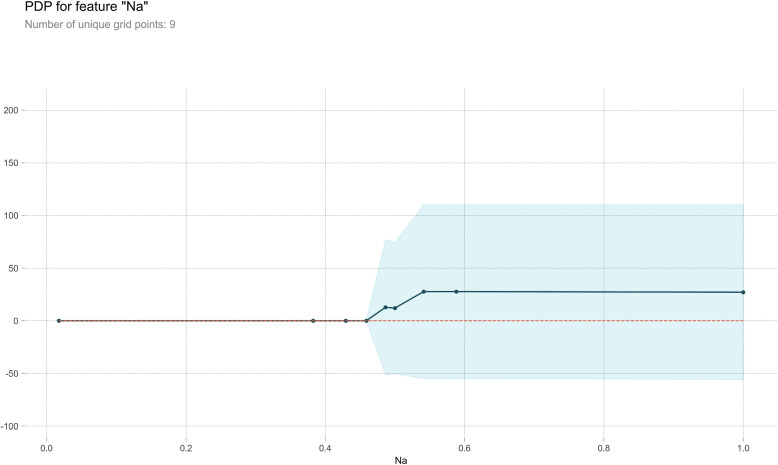
The PDP of indicator Na

**Fig. 12:**
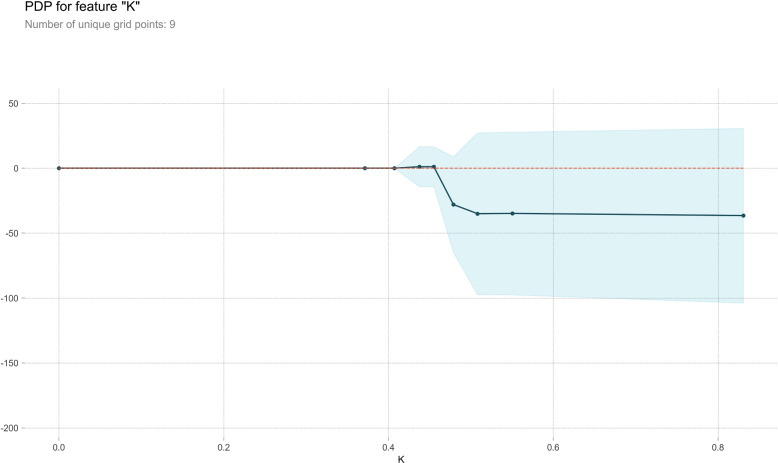
The PDP of indicator K

PDP serves as a post-visual interpretability method, delineating the marginal impact of a specified feature on the anticipated outcome [37]. Within the PDP, the black line epitomizes the alteration in the prediction of bleeding volume, traversing the entire spectrum of conceivable values for the focal indicator while keeping other indicators constant. Six indicators, Ca, HB, WBC, PLT, Na, and K, were meticulously chosen for constructing a comprehensive partial dependence graph, as delineated in Figs. 7, 8, 9, 10, 11 and 12. We kept the other indicators constant to see how these indicators influence the prediction of PPH.

- For the Ca as shown in Fig. 7, When the composition of Ca was less than 0.42, the bleeding volume gradually increased. When the composition of Ca was more than 0.42, the bleeding volume would not change. This prompts us to pay attention to the concentration of Ca ions in the body of delivery women and the remaining risk of calcium deficiency in delivery women.
- For the HB as shown in Fig. 8, When the content of HB is between 0.32 and 0.35, the amount of bleeding will increase accordingly. When the content of HB is less than 0.32 or greater than 0.35, there is almost no change.
- For the WBC as shown in Fig. 9, when the content of WBC is between 0.08 and 0.22, the bleeding will slowly decrease. When the content of HB exceeds 0.22, the amount of bleeding will not change accordingly.
- For the PLT as shown in Fig. 10, when the content of PLT is between 0.4 and 0.45, the bleeding will increase accordingly. When the content of HB exceeds 0.45, the amount of bleeding slowly decreases as it increases.
- For the Na as shown in Fig. 11, When the content of Na is between 0.45 and 0.55, the amount of bleeding will decrease accordingly. When the content of Na is less than 0.45 or greater than 0.55, there is almost no change.
- For the K as shown in Fig. 12, When the content of K is between 0.45 and 0.5, the amount of bleeding will decrease accordingly. When the content of K is less than 0.45 or greater than 0.5, there is almost no change.

PDPs show that these indicators provide important reference values for constructing predictive models. Medical staff can pay attention to these indicators of the delivery women before the cesarean section. As explained in the previous section, the mechanism by which each indicator plays a role in the quantitative predicting model is different. The PDP generated by each indicator is also vastly different.

### Discussion

ML methods, distinguished by the absence of assumptions about input indicators and their relationships with output, offer a compelling advantage through entirely data-driven learning. This feature eliminates the need for rule-based programming, making ML a logical and feasible option for complex predictive tasks. Increasingly, research has focused on applying ML techniques to predict PPH. Various models, such as logistic regression, gradient boosting regressor, XGBoost, and random forest, have been utilized to evaluate the risk of PPH. The gradient boosting regressor reduces prediction errors by combining weak learners, while logistic regression offers insights into the protective or hazardous nature of specific indicators. The XGBoost algorithm is renowned for its fast computation, robust generalization capabilities, and high predictive performance. Similarly, the random forest algorithm, which constructs multiple decision trees, excels in handling large and nonlinear datasets, thereby enhancing accuracy in identifying critical predictive features.

Despite the promising performance observed in prior studies, evidence of the quantitative prediction application of ML in the context of PPH is limited. This study employed six ML methods, including logistic regression, linear regression, gradient boosting regressor, XGBoost, multilayer perceptron, and random forest, to identify the optimal quantitative prediction model for PPH. An interpretable ML-based quantitative prediction of PPH was assessed, with the random forest model outperforming the other algorithms. The MAE of the random forest regression prediction model was 21.7 milliliters, representing a prediction error of less than 5.4$\%$. The RMSE of the random forest regression prediction model was 33.75 milliliters, indicating a prediction error of less than 9.3$\%$. The superior performance of the random forest regression in predicting PPH can be attributed to several reasons, as follows.

- Ensemble Learning: Random forest is an ensemble learning method that combines the predictions of multiple decision trees. By aggregating the predictions of several weak learners (individual decision trees), random forest can mitigate overfitting and improve predictive accuracy.
- Robustness to Noise: Random forest is robust to noisy data and outliers because it uses multiple decision trees. Each tree in the forest is trained on a random subset of the data and features, reducing the impact of individual noisy data points.
- Feature Importance: Random forest measures feature importance, indicating the relative contribution of each input indicator to the predictive performance. This feature selection mechanism helps identify the most relevant predictors of PPH.
- Handling Nonlinearity: Random forest can capture complex nonlinear relationships between input indicators and the target indicator (PPH). This flexibility allows it to effectively model the intricate interactions among various risk indicators associated with PPH.

Additionally, recognizing the general poor interpretability of ML, which may hinder diagnostic strategy formulation by physicians and impede patients’ understanding and cooperation, we employed interpretable ML tools and techniques. Permutation importance analysis highlighted that Ca, HB, WBC, PLT, Na, and K are the top six indicators contributing to the prediction model. Subsequently, PDPs were generated for six selected indicators (Ca, HB, WBC, PLT, Na, and K), providing an intuitive visualization of the impact of each indicator’s change trend on the quantitative prediction of PPH. The key risk indicators identified in this study differ from those highlighted in previous classification prediction models. This discrepancy is due to our focus on quantitative regression prediction, in contrast to the predominantly qualitative classification predictions of earlier research. By explicitly considering the sample size and statistical power, we ensured that our dataset of 6,144 patients was sufficiently robust to detect significant predictors of bleeding volume. It helps enhance the reliability and validity of our findings.

## Conclusions

Based on our self-organized dataset, we applied machine learning methods to quantitatively predict PPH. The random forest method achieved the best performance. And it helped us identify critical predictive indicators. The permutation importance analysis showed that Ca, HB, WBC, PLT, Na, and K were the most critical indicators in predicting PPH. PDPs provided an intuitive visualization of the impact of each indicator’s change trend on the quantitative prediction of PPH.

This study highlights that the random forest model can effectively predict the amount of PPH, providing clinicians with a valuable tool for early intervention. The integration of ML with permutation importance and PDP offers a transparent approach to individual risk prediction, enhancing the safety of cesarean sections and reducing complication rates. By employing a sample size of 6,144, we ensured adequate statistical power to detect significant predictors of bleeding volume, supporting the robustness and reliability of our findings.

Future research will focus on improving ML model accuracy and further exploring the mechanisms behind the identified predictive indicators. Collecting more samples, especially high PPH amounts of samples, is very helpful for model training. Additionally, it will be valuable to study how these important risk indicators affect the quantitative prediction of PPH, thereby providing clinicians with deeper insights into managing and mitigating the risks associated with cesarean sections.

## Data Availability

Data regarding any of the subjects in the study has not been previously published unless specified. The study code was written in Python. All the code in the current study is available from the author on reasonable request by email: wangmeng2003@126.com.
